# Apraxia of Eyelid Opening and Blepharospasm in Two Spinocerebellar Ataxia Type 3 Patients

**DOI:** 10.5334/tohm.677

**Published:** 2022-01-17

**Authors:** Thiago G. Guimarães, Lucas R. Montanha, Fernando Freua, Anderson R. B. Paiva, Lúcia I. Macedo-Souza, Fernando Kok

**Affiliations:** 1 Neurogenetics Unit, Neurology Department, Hospital das Clínicas da Universidade de São Paulo, São Paulo, Brazil

**Keywords:** Eyelid opening apraxia, spinocerebellar ataxia, Blepharospasm

## Abstract

**Background:**

Neuroophthalmological phenotypical particularities of SCA3.

**Phenomenology:**

Eyelid opening apraxia and asymmetrical blepharospasm.

**Educational Value:**

To illustrate the phenomenology for purposes of education.

Spinocerebellar ataxia 3 (SCA3) is an autosomal dominant disorder caused by an abnormal CAG expansion mutation in the *ATXN3* gene, and the most frequent SCA worldwide.

We report the cases of two women with a heterozygous CAG expansion in the *ATXN3* gene (patient 1, 76 CAG repeats; patient 2 with 75 CAG repeats) who have been treated in our outpatient clinic. Their clinical phenotype is remarkable mainly for global ataxia, pyramidal syndrome (mainly on the lower limbs), limb dystonia, and hypokinesia (Video, patient 1, segment 1). Symptoms appeared at 18 years of age in patient 1, and 30 years of age in patient 2.

**Video:** Two SCA3 patients with eyelid opening apraxia and asymmetrical blepharospasm. Patient 1, segment 1 shows severe global hypokinesia during the finger-nose-finger test and severe hands dystonia. Patient 1, Segment 2 shows quite disabling apraxia of eyelid opening and very subtle asymmetrical blepharospasm (worse on the right side). Patient 2, segment 1, focus on observing the asymmetrical blepharospasm (also worse on the right side). Patient 2, segment 2, better elicits the eyelid opening apraxia (less evident in this patient).

Noteworthily, the patients also had eyelid opening apraxia associated with asymmetrical blepharospasm (Video, patient 1, segment 2; patient 2). These findings have already been described in SCA3 and SCA2 patients [1,2]; however, video documentation is hard to find. There is a wide phenotypical overlap between all hereditary dominant ataxias, and particular clinical features may be helpful during the diagnostic workup. Ocular findings may be of major importance to narrow the diagnostic possibilities. Most reviews focus on abnormalities of supranuclear eye movements. For instance, saccadic pursuit, rebound nystagmus, and ophthalmoparesis (mainly vertical), which are prevalent in SCA3 cases [3]. However, both eyelid opening apraxia and blepharospasm seem to receive less attention in most reviews; nonetheless, these appear to be additional clinical findings that points toward both SCA3 and SCA2.

This report aims to draw attention to eyelid opening apraxia and blepharospasm during the neurological examination, and to serve as a source of teaching material to illustrate SCA3’s phenotypical nuances.
